# The association between digital technology use and depression among older people in China: a moderated mediation model

**DOI:** 10.3389/fpsyt.2025.1457967

**Published:** 2025-02-10

**Authors:** Qinmei Wu, Wei He, Jinfu Wang, Litao Du, Xiangli Xue, Qiang He, Yang Pan, Si Chen, Xianliang Zhang

**Affiliations:** ^1^ School of Physical Education, Shandong University, Jinan, China; ^2^ China Institute of Sport Science, Beijing, China; ^3^ School of Physical Education, South China University of Technology, Guangzhou, China; ^4^ School of Nursing and Rehabilitation, Shandong University, Jinan, China

**Keywords:** older people, physical activity, digital technology use, social participation, depression

## Abstract

**Objective:**

To investigate the association among multidimensional (Digital engagement, DE; Digital devices, DD; and Digital purpose, DP) digital technology use and depression in older Chinese, considering social participation as a mediator and physical activity level (PAL) as a moderator.

**Methods:**

Data on 5,744 participants (aged≥60) were extracted from the China Health and Retirement Longitudinal Study 2020 dataset. Depression was assessed using the 10-item Center for Epidemiologic Studies Depression Scale. Logistic regression examined the association between digital technology use and depression. The PROCESS program’s Model 4 evaluated the mediating role of social participation, while Model 7 assessed the moderating role of PAL.

**Results:**

Digital technology use was negatively associated with depression (DE, *OR=* 0.722, 95%CI: 0.609, 0.858; DD, *OR=* 0.739, 95%CI: 0.634, 0.860; DP, *OR=* 0.916, 95%CI: 0.881, 0.952). Various dimensions of digital technology use exerted direct effects on depression scores of 85.87% (DE, *OR=* -0.638, 95%CI: -1.036, -0.240), 86.09% (DD, *OR=* -0.526, 95%CI: -0.844, -0.208), and 87.82% (DP, *OR=* -0.173, 95%CI: -0.259, -0.087), respectively, and social participation mediated 14.13%, 13.91%, 12.18%. PAL exerted a positive moderating effect on the first half path of mediation, especially with the vigorous PAL (DE, *OR=* 0.591, 95%CI: 0.230, 0.952; DD, *OR=* 0.515, 95%CI: 0.206, 0.824; DP, *OR=* 0.157, 95%CI: 0.075, 0.239).

**Conclusions:**

Multidimensional digital technology use showed an association with depression in older adults. Promoting social participation through digital technology use is associated with lower depressive symptom scores, and vigorous PAL further strengthens this association, leading to improved mental health.

## Introduction

The world’s population is aging rapidly. According to the World Health Organization, by 2030, one in six individuals globally will be aged 60 years or older (1). Among this demographic, over 20% of adults aged 60 and above experience psychiatric or neurological disorders (2). Depression is one of the most prevalent mental health disorders. Major depression poses significant threats to individuals’ physical and mental health. Studies have shown that the prevalence rate of depressive symptoms in middle-aged and older adults in China is as high as 20-27% (3). China alone accounts for 17% of the global burden of mental, neurological, and substance use disorders. Furthermore, this burden is estimated to increase by 10% between 2013 and 2025 (4). This situation presents a significant challenge for global public health.

Many studies have investigated factors associated with depression in the past (5–7). Researchers have analyzed and identified several potential risk factors for depression, such as female gender, family relationships, medical conditions (7), advanced age, and years of education (5). Enhancing social health and improving mental well-being are critical in identifying risk factors for depressive symptoms, and most previous studies have concentrated on this aspect. However, both preventing the onset of depression and alleviating its symptoms require further exploration of the potential protective factors associated with depression.

Digital technology use is increasingly central to mobile health (mHealth) and facilitates the adaptation of older adults to the big data-driven health environment (8). Numerous studies have identified the benefits of digital technology use for the physical and mental health of older adults. For instance, it can alleviate depressive symptoms (9) and enhance the overall happiness of older adults (10). In this regard, Network Gain Effect theory explains that Internet use can enhance individuals’ subjective class identity by increasing their cultural practices and capital (11). This positive identification improves individuals’ social adaptability, accelerates their role-reconfiguration process, and leads to more positive emotional experiences. Additionally, digital technology use can benefit older adults by strengthening their objective ties to society and increasing the frequency of social interactions. According to Social Support theory, individuals can obtain emotional support and practical assistance, through interactions and connections facilitated by digital technology use, and thus maintain or improve their mental health (12). Conversely, Information Depression theory posits that individuals’ exposure to numerous negative news reports on the Internet can result in a sustained decline in social trust and a decreased willingness to engage in social activities, ultimately adversely affecting their mental health (13). Similarly, Substitution Effect theory explains the negative impacts of digital technology use. Internet use has been associated with heightened levels of depression and loneliness, potentially due to diminished communication with family members and a reduction in the size of social networks (14). Therefore, it is evident that “social participation” plays an important role in the association between digital technology use and depression, although the exploration of the association between digital technology use and social participation has not reached a consistent conclusion.

Physical activity (PA) is a crucial factor influencing healthy aging. In recent years, there has been increasing attention on the associations among digital technology use, PA and social participation. For the association between digital technology use and PA, some studies indicate that social media use may dampen efforts to increase PA (15, 16). Long-term engagement with social media and electronic games may lead to the adoption of a sedentary lifestyle by individuals. However, it has also been shown that digital technology use can promote PA participation (17). Social Cognition theory (18) explains the differences in these results. In the digital era, people can access a wealth of sports-related information through digital media, which can encourage participation in physical activity. Nevertheless, individuals’ choices are influenced by their social environments and the behaviors of others, suggesting that the impact of digital technology use may vary. In addition, PA also has an impact on social participation. Positive Emotion Expansion theory (19) posits that individuals who experience positive emotions from PA can expand their social resources and reduce feelings of isolation. Furthermore, the social participation and communication that arise from PA can further mitigate depression. For example, research indicated that PA can enhance social participation and foster the development of social support networks (20), it may serve as a potential strategy to prevent social isolation and loneliness. Therefore, the differences in the association between digital technology use and PA may also influence the association between PA and social participation, as well as the association between digital technology use and social participation.

At the same time, the benefits of physical activity on depression in older adults have been revealed. The findings suggested a negative association between PA and the risk of depressive symptoms although gender may be a key factor in PA influencing depression (21). Additionally, studies have suggested that interactive social media interventions may increase physical activity, thereby potentially improving well-being scores (22). The use of the Internet can be combined with offline factors such as exercise and interaction with others, having a positive impact on mental health (23). What’s more, the frequency of physical activity may partially mediate the relationship between Internet use and depressive symptoms in older adults to some extent (24). Therefore, we speculate that there may be a moderating effect on digital technology use, physical activity, and depression. However, focusing solely on the frequency of PA does not adequately explain this association in terms of physiological mechanisms. We need to further consider total physical activity versus level.

Hence, it is hypothesized that the level of PA (PAL) may also play an important role in the association among digital technology use, social participation, and depression. However, the role of PAL in the association between multi-dimensional digital technology use, social participation, and depression has not been extensively examined. The association between these four variables remains unclear. Based on the above, we aim to further investigate how PA and social participation influence the association between digital technology use and depression among older adults.

**Figure 1:**
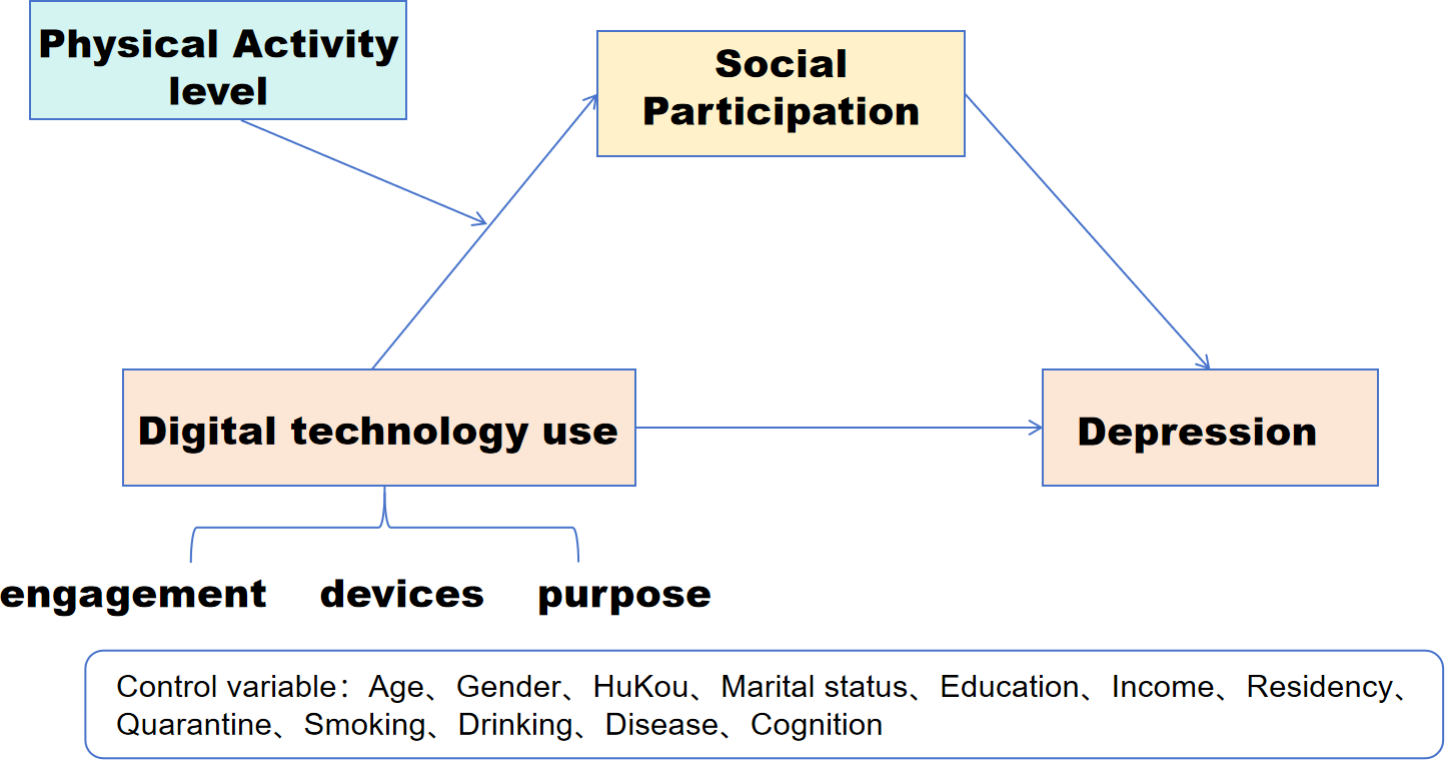
Hypothesized moderated mediation model.

To facilitate the above examinations, the study devised a moderated mediation model (see **Figure 1**) and presented the following hypotheses:

Hypothesis 1. digital technology use of each dimension (digital engagement, DE; digital devices, DD; and digital purpose, DP) would negatively predict depression of older adults.

Hypothesis 2. Social participation would mediate the association between digital technology use and depression.

Hypothesis 3. Physical activity level (PAL) would positively moderate the association between forwarding digital technology use and social participation.

## Methods

### Sample and data source

China Health and Retirement Longitudinal Study (CHARLS) is a nationally representative, large-scale population follow-up survey conducted across 28 provinces in China. It started in 2008 and conducted four national tracking surveys in 2011, 2015, 2018 and 2020. The survey, employing a multi-stage probability-sampling method, collects comprehensive data regarding family demographics, health status, and other pertinent variables for scientific research aimed at addressing the needs of middle-aged and older individuals. Given the rapid growth in Internet use, this study utilizes cross-sectional data from the latest fifth-wave survey conducted in 2020.

**Figure 2:**
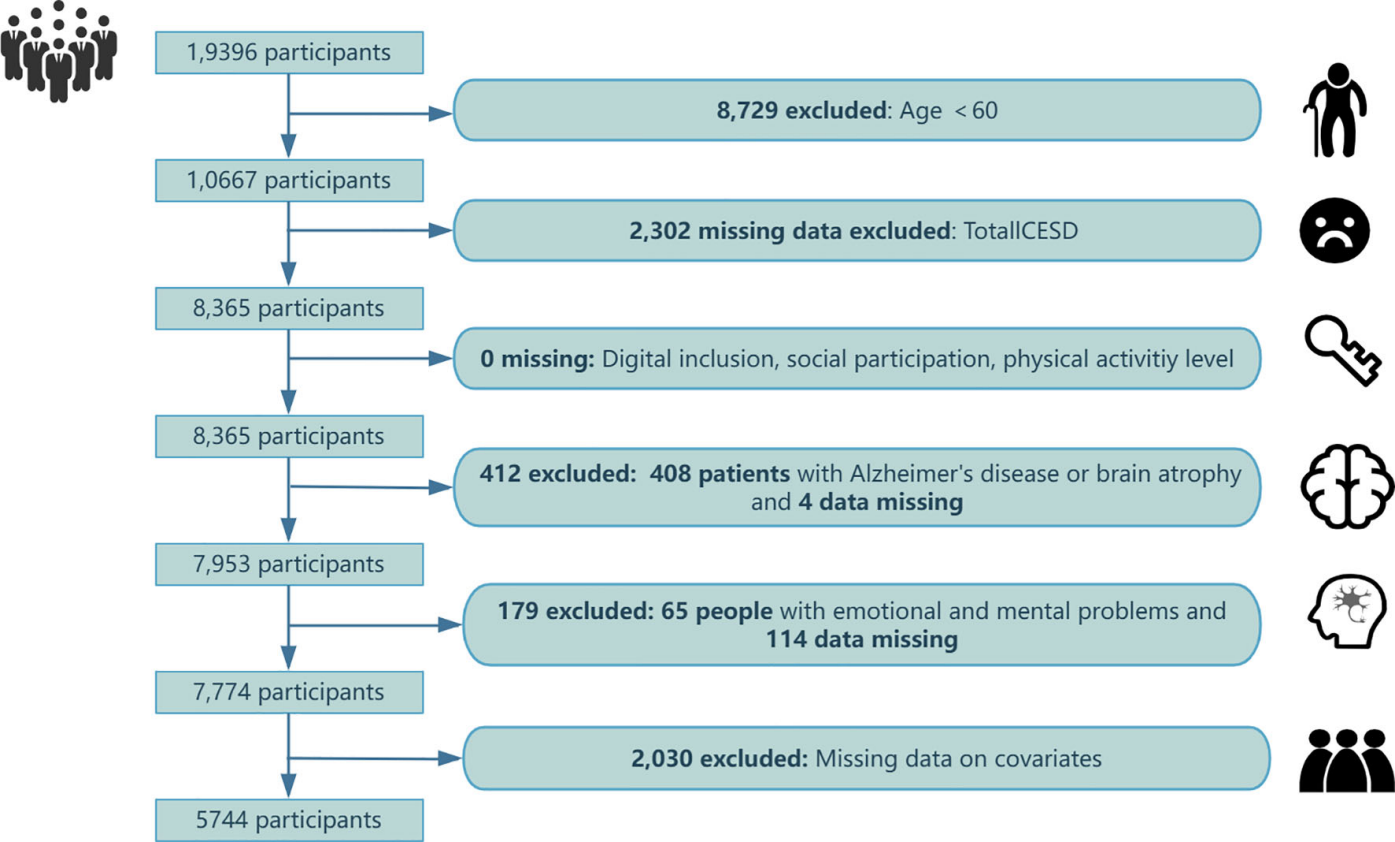
Data exclusion process.

Following the initial screening of the raw data, we performed the sample exclusion based on the exclusion criteria, and missing values for other covariates were also deleted. In our analysis, only samples with complete information were retained. **Figure 2** shows the detailed data exclusion process. To investigate the association between physical activity, social participation, digital technology use, and depression in older adults, we selected 5,744 respondents based on the following criteria: I. Full information on digital technology use, social participation, physical activity, the 10-item Center Depression Scale (CES-D-10) score and covariates; II. Age≥60. Excluded: I.Age<60; II. Individuals with Alzheimer’s disease (AD) and other mental illnesses.

Before initiating the CHARLS survey, trained interviewers informed participants of the survey content, and all participants provided written informed consent. The Ethics Review Board of Peking University has granted ethical approval for all waves of the CHARLS study (IRB 00001052-11015).

### Depression

The investigators used the CES-D-10 to assess the respondents’ feelings and behaviors during the previous week and thus their depressive symptoms. Studies have demonstrated that the Cronbach coefficient of CES-D 10 is 0.87, which has high validity and reliability in the Chinese middle-aged and older adult population (25). CES-D-10 responses were scored on a 4-level scale: 0= <1 day; 1 = 1-2 days; 2 = 3-4 days; 3 = 5-7 days. The fifth question was scored in reverse with the eighth question. Respondents had depression scores ranging between 0 and 30 (26). Lower scores indicate lower levels of depression and vice versa. Furthermore, the threshold 12 was commonly used to identify depression, and studies demonstrated that this threshold was effective in identifying clinically significant depression (27, 28). Therefore, this study established a dichotomous variable at the 12-point point to determine whether respondents had depression (0= no, 1= yes) and those with scores greater than or equal to 12 were considered as having depression. The CES-D-10 score was used for mediation and moderation analyses to examine changes in depression under different mechanisms of action.

### Digital technology use

This study aimed to assess the multidimensional digital technology use of Chinese older adults in the following aspects: whether to engage the Internet, the diversity of device use, and the diversity of digital purposes. Asked “Have you used the internet in the past month?” to determine digital engagement (DE, dichotomous variable: 0=Not involved, 1=Participate). Next, inquired “What devices do you utilize to access the Internet?” to assess older adults’ digital device usage (DD, continuous variable, ranging from 0-5, where points are awarded for desktop computers, laptops, tablets, mobile phones, and other digital devices. Higher scores indicated greater participation in digital devices). Finally, questions such as “What are your typical online activities?”, “Do you engage in mobile payments, such as with Alipay, WeChat Wallet, etc.?”, “Are you familiar with using WeChat?”, and “Do you post updates on WeChat Moments?” evaluated the digital purpose (DP) of older adults (continuous variable, ranging from 0-8, covering chatting, news, videos, games, financial management, mobile payments, WeChat, and social media interactions. A higher score reflected a more diverse digital technology use).

### Physical activity level

The survey was divided into 3 physical activity types. I. Vigorous intensity physical activity (VPA): such as digging, farming, aerobic exercise; II. Moderate intensity physical activity (MPA): such as mopping, Taijiquan, brisk walking, etc.; III. Low intensity physical activity (LPA): such as walking, etc. According to the CHARLS questionnaire, the daily duration of physical activity types is divided into 5 categories (0 min-10min, 10-29 min, 30-119 min, 120-239min, ≥240 min), and the exercise time of VPA, MPA and LPA is calculated using intermediate values (29). Weekly physical activity duration=number of days of physical activity type×duration of physical activity type per day. The amount of PA is calculated using metabolic equivalent (MET). According to the International Physical Activity Questionnaire (IPAQ) criteria, low intensity MET was assigned 3.3, moderate intensity MET 4.0, and vigorous intensity MET 8.0. Thus, total PA = 8.0×total weekly duration of VPA + 4.0×total weekly duration of MPA + 3.3×total weekly duration of LPA (30). Weekly PAL was divided into low physical activity level (LPAL, <600 METs/week), moderate physical activity level (MPAL, 600-3,000 METs/week) and vigorous physical activity level (VPAL, > 3,000 METs/week) (29).

### Social participation

To measure social participation, we assessed 8 types of social activity participation in the CHARLS 2020 data set by points 0 to 3. A score of 0 signifies no participation, 1 infrequent participation, 2 almost weekly participation, and 3 almost daily participation. These activities include: I. Visiting and interacting with friends. II. Playing games like mahjong, chess, and cards in the community activity room. III. Assisting relatives, friends, or neighbors. IV. Participating in dance, fitness, and qigong activities. V. Attending community events. VI. Volunteering or engaging in charity work. VII. Providing care for patients or the disabled, or attending school/training courses. VIII. Other social activities. Finally, the total score was calculated, ranging between 0 and 24 (31). The higher the score, the greater the social participation (32).

### Covariates

Based on previous studies (33, 34), this study considered potential confounding variables related to digital technology use, social participation, physical activity, and depression, controlling for age, gender, HuKou, marital status, education level, income, residency, smoking, drinking, diseases and cognitive scores. Cognitive scores were calculated using the Telephone Interview Scale for Cognitive Status (TICS) (35), which consists of 3 dimensions: mental psychological state, immediate recall ability, and delayed recall ability. I. Mental state: the research subjects were asked to answer the current year, month, day, weekday, and season, and to calculate 5 times consecutive minus 7 (star with 100). II. Immediate recall ability: read 10 words to the research subject, after the number of correct recalls is the score. III. Delayed recall: after some time, the respondents were asked to recall the previous 10 words. One point for each question, the total score of the scale is 30 points, the higher the score, the better the cognitive function. In addition, considering that this data comes from the COVID-19 outbreak in 2020, the quarantine was also controlled. Specific assignment instructions for each covariate are detailed in **Table 1**.

**Table 1:** Description of the specific assignment of the covariates.

Control variable description	
Age	Continuous variable
Gender	1= Male, 2= Female
HuKou	1= Agriculture, 2= Non-agricultural, 3= Unified HuKou, 4= Don’t have HuKou.
Marital status	1= Married and living with a spouse; 2= Married, but he did not live with his spouse because of work and other reasons; 3=Separation (no longer living together as a spouse); 4= Divorce; 5= Widowed, 6= Never married.
Education	1= Illiterate, 2= Primary school, private school and below, 3= Middle school (junior high school, high school, vocational school), 4= College degree or above
Income	1= Yes, 2= No
Residency	1= Urban center, 2= Urban rural township junction, 3= Rural, 4= Others
Quarantine status	1= Yes, 2= No
Smoking	1= Yes and still, 2= Yes but quit, 3= Never
Drinking	1= More than once a month, 2= Less than once a month, 3= Never
Cognition	Continuous variable
Diseases^a^	0= None, 1 = 1 type, 2 = 2 or more types

^a^Diseases are hypertension, hyperlipidemia, hyperglycemia, malignant tumors, chronic lung disease, liver disease, heart disease, stroke, kidney disease, stomach disease, arthritis, asthma, parkinsonismus, mental problems, and memory-related diseases.

### Statistical analysis

We used Stata MP 17.0 and IBM SPSS Statistics 27 to clean, merge, and statistically analyze data. Data were tested for normality using histograms. Continuous data are expressed as the mean (SD). Categorical data are expressed as frequency and percentage. Differences between the groups were tested by Kruskal-Wallis H test and chi-square test. Use Spearman correlation analysis to assess the correlation between variables. Logical regression was used to examine the association between digital technology use and depression in older adults. Three models were used to adjust for confounders. Model 1 adjusted for gender and age. Model 2 additionally adjusted for Model 1 variables plus HuKou, marriage, education, diseases, income, smoking, drinking, residency, and quarantine. Model 3 additionally adjusted for Model 2 variables plus cognitive score.

We tested the variance inflation factor (VIF) between digital technology use, social participation, PAL and depression. The VIF was all below 2, indicating that there is no multicollinearity between them. To verify the robustness of our findings, we conducted subgroup analyses by age (24, 36, 37), gender (38), and chronic disease status (24) to explore the association between digital technology use and depression. At this time, we divided the age into three groups: 60-69, 70-79, and ≥80. Moreover, an exploratory factor analysis of the raw data using Harman’s single factor test revealed that 16 factors had characteristic roots greater than 1, with the first common factor explaining 15.137% of the total variance, which is less than the critical value of 40%. Therefore, there is no serious problem of common methodological bias in this study.

Furthermore, the mediating role of social participation between digital technology use and depression was examined using Model 4 of the PROCESS macro developed by Hayes (39). The mediating effect analysis was conducted using digital technology use of each dimension as the independent variable (X), depression score as the dependent variable (Y), social participation as the mediating variable (M), and other factors as covariates. The principle of the mediating effect is adopted from the Boostrap sampling method of the products of coefficients (test the significance of a*b; a, b, meanings are shown in the **Appendix-Supplementary Figure**). The 95% confidence interval (CI) was estimated via 5000 bootstrap replications, and the mediation effect was deemed statistically significant if the 95% CI did not contain 0. To the mediating effect, we added the moderator variable (W) PAL. Model 7 examined the moderating effect of PAL, centralizing the variables prior to adjustment. We adjusted for gender and age, HuKou, marriage, education, diseases, income, smoking, drinking, residency, and quarantine, cognitive scores both in Model 4 and Model 7. The significance level was set at α = 0.05.

## Results

### Descriptive results for the study variables

The final analysis encompassed data from 5,744 older adults, averaging 68 years old. Among these, 878 older adults with LPAL, 1,703 with MPAL, and 3,163 with VPAL, indicating more older adults with VPAL. In addition, among these factors (**Table 2**), the gender, number of chronic diseases and quarantine status between individuals did not differ significantly between PAL.

**Table 2:** Analysis of the description of the demographic characteristics.

Variables	Mean (± SD)/n (%)				P
	Total	LPAL	MPAL	VPAL	
	(n=5,744)	(n=878)	(n=1,703)	(n=3,163)	
**Age (years)**	67.64 (5.77)	68.94 (6.43)	68.90 (6.05)	66.92 (5.30)	<0.001^a^
Gender, n (%)					
Male	3,178 (55.33)	464 (52.85)	957 (56.19)	1,757 (55.55)	0.251^b^
Female	2,566 (44.67)	414 (47.15)	746 (43.81)	1,406 (44.45)	
HuKou					
Agricultural	3,956 (68.87)	647 (73.69)	999 (58.66)	2,310 (73.03)	<0.001^b^
Non-Agricultural	1,066 (18.56)	126 (14.35)	432 (25.37)	508 (16.06)	
Unified residence	720 (12.53)	104 (11.85)	271 (15.91)	345 (10.91)	
Do not have HuKou	2 (0.03)	1 (0.11)	1 (0.06)	0	
Marital status					
Married and Living with a spouse	4,484 (78.06)	648 (73.80)	1,309 (76.86)	2,527 (79.89)	<0.001^b^
Married but not Living with spouse temporarily	251 (4.37)	23 (2.62)	76 (4.46)	152 (4.81)	
Separated	22 (0.38)	9 (1.03)	5 (0.29)	8 (0.25)	
Divorced	56 (0.97)	4 (0.46)	21 (1.23)	31 (0.98)	
Widowed	927 (16.14)	194 (22.10)	290 (17.03)	443 (14.01)	
Never married	4 (0.07)	0	2 (0.12)	2 (0.06)	
Education					
Illiterate	960 (16.71)	151 (17.20)	209 (12.27)	600 (18.97)	<0.001^b^
≤Primary school	2,820 (49.09)	460 (52.39)	806 (47.33)	1,554 (49.13)	
Middle/High school	1,848 (32.17)	257 (29.27)	639 (37.52)	952 (30.10)	
≥University	116 (2.02)	10 (1.14)	49 (2.88)	57 (1.80)	
Income					
Yes	897 (15.62)	110 (12.53)	582 (18.40)	582 (18.40)	<0.001^b^
No	4,847 (84.38)	768 (87.47)	2,581 (81.60)	2,581 (81.60)
Residency					
Urban center	1,552 (27.02)	192 (21.87)	636 (37.35)	724 (22.89)	<0.001^b^
Urban-rural integration	642 (11.18)	103 (11.73)	221 (12.98)	318 (10.05)	
Rural	3,544 (61.70)	583 (66.40)	842 (49.44)	2,119 (66.99)	
Others	6 (0.10)	0	4 (0.23)	2 (0.06)	
Quarantine					
Yes	70 (1.22)	7 (0.80)	16 (0.94)	47 (1.49)	0.118^b^
No	5,674 (98.78)	871 (99.20)	1,687 (99.06)	3,116 (98.51)	
Types of chronic diseases					
No	3,654 (63.61)	565 (64.35)	1,055 (61.95)	2,034 (64.31)	0.531^b^
1 type	1,418 (24.69)	212 (24.15)	435 (25.54)	771 (24.38)	
≥2 types	672 (11.70)	101 (11.50)	213 (12.51)	358 (11.32)	
Smoking					
Yes (still)	1,601 (27.87)	245 (27.90)	448 (26.31)	908 (28.71)	0.001^b^
Yes (quit)	1,052 (18.31)	179 (20.39)	355 (20.85)	518 (16.38)	
Never	3,091 (53.81)	454 (51.71)	900 (52.85)	1,737 (54.92)	
Drinking					
Usually	1,628 (28.34)	223 (25.40)	460 (27.01)	945 (29.88)	<0.001^b^
Occasionally	545 (9.49)	60 (6.83)	158 (9.28)	327 (10.34)	
Never	3,571 (62.17)	595 (67.77)	1,085 (67.77)	1,891 (59.79)	
Depressive symptoms					
Yes	1,564 (27.23)	283 (32.23)	409 (24.02)	872 (27.57)	<0.001^b^
No	4,180 (72.77)	595 (67.77)	1,294 (75.98)	2,291 (72.43)	
**Cognitive scores**	15.79 (4.75)	14.96 (4.85)	16.33 (4.75)	15.72 (4.68)	<0.001^a^

^a^Kruskal-Wallis H test.

^b^Chi-square test.

LPAL, low physical activity level; MPAL, moderate physical activity level; VPAL, vigorous physical activity level.

### Correlation analysis

The results of the correlation analysis (**Table 3**) showed that digital participation, social participation, and PAL were all significantly negatively associated with depression scores, and the significant correlation between the main variables indicated that the mediation could be further tested.

**Table 3:** Spearman correlation analysis.

	Depression	DE	DD	DP	SP	PAL
Depression	1.000	-0.164**	-0.170**	-0.174**	-0.070**	-0.038**
DE		1.000	0.994**	0.985**	0.219**	0.031*
DD			1.000	0.983**	0.221**	0.033*
DP				1.000	0.232**	0.035**
SP					1.000	0.078**
PAL						1.000

*P<0.05, **P<0.01.

DE, digital engagement. DD, digital devices. DP, digital purpose. SP, social participation. PAL, physical activity level.

### Multidimensional digital technology use and depression in older adults

**Figure 3:**
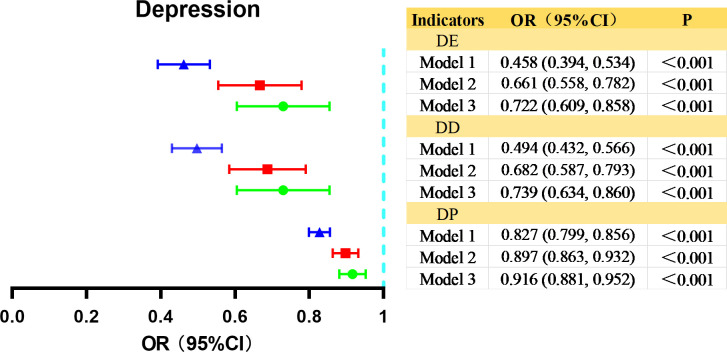
Logistic regression analysis of the association between multidimensional digital technology use and depression in older adults. DE, digital engagement. DD, digital devices. DP, digital purpose. Model 1, gender and age were adjusted. Model 2 additionally adjusted for Model 1 variables plus HuKou, marriage, education, diseases, income, smoking, drinking, residency, and quarantine. Model 3 additionally adjusted for Model 2 variables plus cognitive score.

We created dichotomous variables based on depression scores (with a cutoff of 12) to examine the association between digital technology use and depression in the entire sample using logistic regression (**Figure 3**). Model 1 found that digital technology use of all dimensions was negatively associated with depression. This association remained significant after full adjustment (Model 3, DU: 0.722, 95%CI: 0.609, 0.858; DD: 0.739, 95%CI: 0.634, 0.860; DP: 0.916, 95%CI: 0.881, 0.952). This suggests that digital technology use is independently associated with depression. Hypothesis 1 was supported.

### The subgroup analysis

To verify the robustness of the results, we analyzed the different subgroups based on model 3 to explore the association between digital technology use and depression (details are provided in **Appendix-Supplementary Table**).

Results showed a negative association between digital technology use and depression in male (DE: *OR=* 0.664, p= 0.001; DD: *OR=* 0.694, p= 0.001; DP: *OR=* 0.913, p= 0.001), along with similar results in female (DE: *OR=* 0.786, p= 0.053[marginal significance]; DD: *OR=* 0.791, p= 0.030; DP: *OR=* 0.922, p= 0.004). Across different age subgroups, digital technology use was inversely associated with depression in the 60-69 age groups (DE: *OR=* 0.737, p= 0.002; DD: *OR=* 0.750, p= 0.001; DP: *OR=* 0.921, p< 0.001), while no clear association was observed in the other age groups. Furthermore, digital technology use was also negatively associated with depression in the subgroup of older adults without disease (DE: *OR=* 0.740, p= 0.008; DD: *OR=* 0.017, p= 0.017; DP: *OR=*0.914, p= 0.001) or with one chronic disease (DE: *OR=* 0.642, p= 0.011; DD: *OR=* 0.614, p= 0.002; DP: *OR=* 0.898, p= 0.006), which was not found in older adults with two or more chronic conditions.

### Mediation analyses

**Figure 4:**
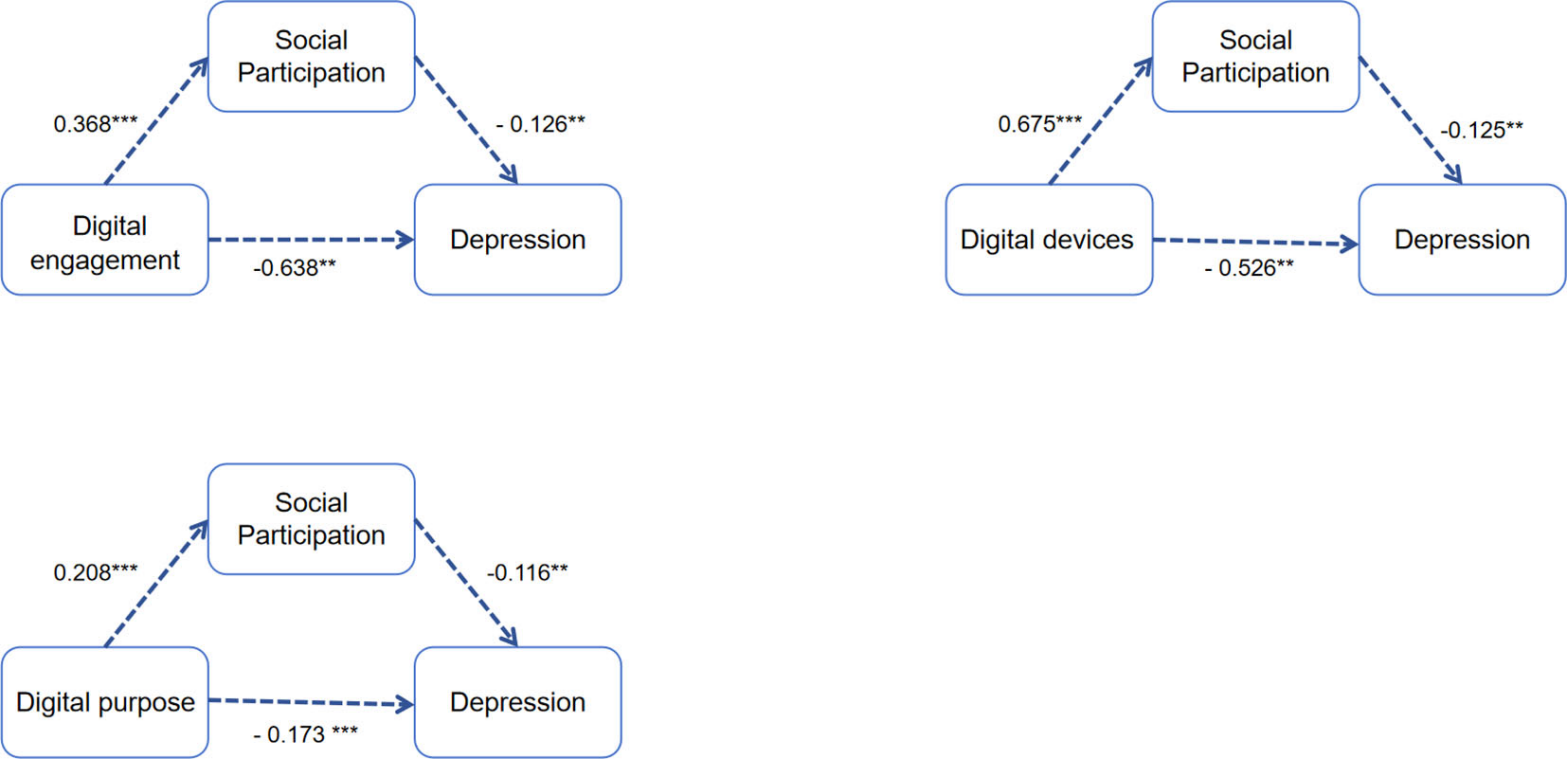
Examination of the mediation model. **P<0.01, ***P<0.001.

The results indicated that digital technology use in each dimension significantly positively predicted social participation and significantly negatively predicted depression scores. Digital engagement, diversity of digital devices, and diversity of digital purpose had direct effects on depression scores of 85.87% (B= -0.638, 95%CI: -1.036, -0.240), 86.09% (B= -0.526, 95%CI: -0.844, -0.208), and 87.82% (B= -0.173, 95%CI: -0.259, -0.087), respectively. Social participation mediated the remaining 14.13%, 13.91%, and 12.18%. This suggests that older adults can maintain lower levels of depression by increasing social participation. Hypothesis 2 was supported. As shown in **Table 4**, **Figure 4**.

**Table 4:** The mediating effect of social participation in the association between digital technology use and depression among older adults.

		Effect	se	t	p	LLCI	ULCI	Effect%
DE	Total effect	-0.743	0.200	-3.714	<0.001	-1.136	-0.351	
	Direct effect	-0.638	0.203	-3.146	0.002	-1.036	-0.240	85.87%
	Indirect effect	-0.105	0.034			-0.173	-0.040	14.13%
DD	Total effect	-0.611	0.160	-3.814	<0.001	-0.924	-0.297	
	Direct effect	-0.526	0.162	-3.241	0.001	-0.844	-0.208	86.09%
	Indirect effect	-0.085	0.027			-0.138	-0.034	13.91%
DP	Total effect	-0.197	0.043	-4.591	<0.001	-0.281	-0.113	
	Direct effect	-0.173	0.044	-3.953	<0.001	-0.259	-0.087	87.82%
	Indirect effect	-0.024	0.008			-0.040	-0.008	12.18%

DE, digital engagement. DD, digital devices. DP, digital purpose.

### Moderating effect of the PAL

To test the moderating role of PAL in the association between digital technology use on social participation and depression in older adults, this study divided PAL into three categories: low, medium and high according to the moderated mediating effect test. Model 7 in the PROCESS was used to test the moderating effect of different PALs.

The results show that PAL moderates the first half of the pathway of this mediated process (**Table 5**). Hypothesis 3 was supported. VPAL could positively moderate the association between digital technology use and social participation in various dimensions (DE: 0.591, 95%CI: 0.230, 0.952; DD: 0.515, 95%CI: 0.206, 0.824; DP: 0.157, 95%CI: 0.075, 0.239), and showed significant difference from LPAL and MPAL.

**Table 5:** Moderated mediation effect test.

	coeff	se	t	p	LLCI	ULCI
Constant	-0.958	0.397	-2.412	0.016	-1.736	-0.179
DE	0.446	0.171	2.613	0.009	0.111	0.781
MPAL	0.436	0.089	4.910	<0.001	0.262	0.610
VPAL	0.440	0.080	5.477	<0.001	0.282	0.597
DE×MPAL	0.103	0.194	0.529	0.597	-0.278	0.483
DE×VPAL	0.591	0.184	3.212	0.001	0.230	0.952
Constant	-0.697	0.392	-1.778	0.075	-1.466	0.072
DD	0.319	0.149	2.142	0.032	0.027	0.611
MPAL	0.475	0.080	5.913	<0.001	0.317	0.632
VPAL	0.594	0.074	8.010	<0.001	0.449	0.739
DD×MPAL	0.099	0.166	0.594	0.553	-0.227	0.424
DD×VPAL	0.515	0.158	3.267	0.001	0.206	0.824
Constant	-0.820	0.391	-2.099	0.036	-1.585	0.054
DP	0.101	0.040	2.548	0.011	0.023	0.178
MPAL	0.468	0.080	5.837	<0.001	0.311	0.625
VPAL	0.592	0.074	7.994	<0.001	0.447	0.738
DP×MPAL	0.037	0.044	0.848	0.397	-0.049	0.123
DP×VPAL	0.157	0.042	3.735	<0.001	0.075	0.239

DE, digital engagement. DD, digital devices. DP, digital purpose.

LPAL, low physical activity level. MPAL, moderate physical activity level.VPAL, vigorous physical activity level.

**Figure 5:**
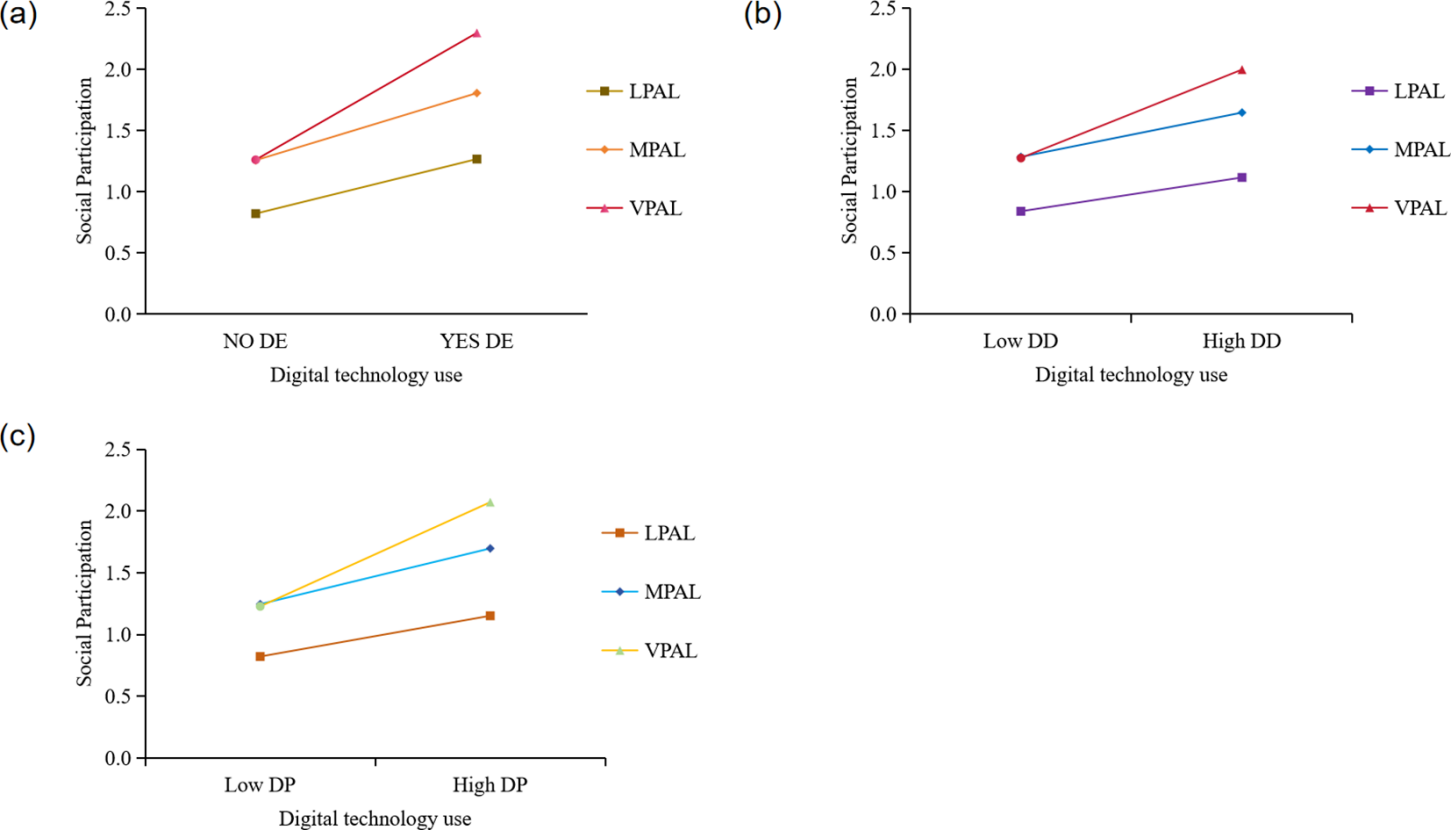
Simple slope analysis. DE, digital engagement. DD, digital devices. DP, digital purpose. LPAL, low physical activity level; MPAL, moderate physical activity level; VPAL, vigorous physical activity level. **(A)** The moderating role of physical activity levels between digital engagement and social participation. **(B)** The moderating role of physical activity levels between the use of digital devices and social participation. **(C)** The moderating role of physical activity levels between digital purpose and social participation.

A simple slope analysis was further performed, as shown in **Figure 5**. The results indicate that digital technology use is significantly associated with positive social participation at any PAL. As the digital technology use improves, the increase in social participation among the VPAL population was greater than that in MPAL than in LPAL (LPAL [DE, β: 0.446, t=2.613, 95%CI: 0.111, 0.781; DD, β: 0.319, t=2.142, 95%CI: 0.027, 0.611; DP, β: 0.101, t=2.548, 95%CI:0.023, 0.178]; MPAL [DE, β: 0.549, t=5.353, 95%CI: 0.348, 0.750; DD, β: 0.418, t=5.061, 95%CI: 0.256, 0.579; DP, β: 0.138, t=6.476, 95%CI: 0.096, 0.180]; VPAL [DE, β: 1.037, t=12.717, 95%CI: 0.877, 1.197; DD, β: 0.834, t=13.099, 95%CI: 0.709, 0.959; DP, β: 0.258, t=14.940, 95%CI: 0.224, 0.292]).

## Discussion

This study aimed to examine the associations of different PALs and social participation on the association between multidimensional digital technology use and depression in older adults. This study concludes that digital technology use across all dimensions is significantly negatively associated with depression and with lower depression levels by enhancing social participation. Furthermore, our additional moderated mediation model demonstrated that PAL has a positive moderating effect on social participation. VPAL was found to be more effective and significantly different from both LPAL and MPAL.

In the digital era, an increasing number of studies have explored the association between Internet engagement and mental health of older adults. Digital technology has spawned information-rich media, with each mode of digital technology use serving distinct function, and “whether or not to engage Internet” cannot be the sole determinant of mental health. Particularly in health research, a multidimensional analysis is essential, transcending a simple binary assessment of Internet usage among older adults (40). Therefore, in addition to examining the association between whether older adults use the Internet and depression, we also examined the variety of digital device usage and the range of digital purposes. The results showed that digital technology use across all dimensions—spanning Internet engagement and usage, the number of digital devices employed, or the diversity of digital purposes—was independently associated with depression. Notably, DP showed a more significant protective effect against depression. Older adults with a more diverse range of DP tend to achieve effective digital inclusion, including posting on WeChat Moments, browsing TikTok, engaging in online communication, making electronic payments and so on. This enhances their connectivity with the information society, leading to the accumulation of social capital, information acquisition, and recreational engagement (23). It also makes them less prone to social alienation and more likely to gain enhanced life experiences and emotional well-being. DE and the diverse DD do not sufficiently demonstrate this capability. Typically, the majority of older individuals use various digital devices for similar purposes. For instance, they might use smartphones, tablets, and computers primarily for video consumption. Consequently, this could limit the avenues for obtaining information and emotional support.

Furthermore, our subsequent subgroup analysis suggested potential heterogeneity in these results. Overall, the negative association between digital technology use and depression was significant for both men and women (with women showing marginal significance in DE). Moreover, the results showed that digital technology use was negatively associated with depression exclusively among the elderly population aged 60-69, whereas no clear association was observed in the older age group (≥70). A study by van Boekel et al. found that older adults younger than 70 years devoted more time to the Internet, frequented the Internet more often, and engaged in a broader array of Internet activities compared to adults older than 70 years of age (41). Therefore, we surmise that the reason for this difference is that younger older adults are more digitally adept and can derive greater benefits through the Internet. Another finding of our study was that the negative association between digital technology use and depression was more pronounced among older adults with no chronic disease or only one chronic disease. Coincidentally, Liu, Z et al. found in a heterogeneity analysis that the positive impact of digital engagement on alleviating depression was more pronounced among older adults without chronic conditions (24). This finding provides evidence for the results of the present study. The explanation for this is that older adults with more chronic illnesses tend to have a deteriorated mood state, may focus more attention on their health status and the pain associated with their illness, and may possess a lower motivation to live and experience social avoidance. Thus, the mitigating effect of digital technology use on depression is relatively weak in older adults with chronic conditions.

Moreover, some studies hold different attitudes toward the association between digital technology use and depression. For example, Petkovic J et al. did not find any association between digital usage and mental health (22). They believe that using the Internet alone does not improve the mental health of older adults. But when the Internet provides psychological help to older adults or links exercise and social interaction, it can play a positive role in alleviating depression. Alternatively, Kraut et al. proposed that the use of the Internet could lead to increased depression and loneliness because it lowers communication with family members and lowers the size of social circles (14). This reminds us that social participation may be key to explaining differences in the association between digital technology use and depression in older adults.

Based on this, we further examined the association between social participation in digital technology use and depression. Our results indicated that social participation mediates the association between digital technology use and depression in various dimensions. Digital technology use was positively associated with social participation. This finding was in accord with prior research (33, 42–44). As for the explanation of the association between digital technology use, social participation, and depression, Network Gain Effect theory and Substitution Effect theory are mostly discussed. This study supports the former. We believe that digital technology use can strengthen the objective connection between older adults and society, the abundance and difference of daily information sources are beneficial to people’s psychology, and the Internet can break the barriers of time and space, expand the breadth of people’s social interaction, and then have a positive “spillover effect” on social participation. From a psychosocial perspective, their social networks become smaller due to work, family, physical conditions and other factors. The Internet can overcome social and spatial barriers and become a convenient way to help them stay connected with the outside world (45). Digital technology use enhances older adults’ sense of belonging or control and greater self-esteem by promoting social participation (46). This may lead to better mental health and the alleviation of depression. From a neurological perspective, the entertainment information or favorite information that the elderly can access when engaging in social activities through digital technology use, such as getting more “likes” on social media. At this point, the reward circuitry (ventral tegmental area) may be more activated, leading to the release of dopamine (47). This may induce a pleasant response and reduce depressive symptoms.

In contrast, some studies support Substitution Effect theory (48). The reason for this outcome gap may be the different degrees and frequencies of digital technology use. They believe that there is a significant negative correlation between digital engagement and mental health. This is probably because excessive use of the Internet erodes people’s physical interactions, disconnects them from reality, cuts off established social associations, and increases the risk of psychological problems (49). However, in 2023, only 15.6% of Chinese Internet users were over the age of 60 (50), and only half of these online users were able to master one digital skill (51). Therefore, we speculate that Internet addiction in older adults is not very likely, and the Internet can be a positive means to maintain their existing social contacts and establish new ones, help and promote their participation in other offline activities and social participation, and thus bring about a realistic experience with positive effects on the mental health of older adults.

The boundaries between physical activity and social participation are often blurred, and the two activities often intertwine and influence each other in the daily activities of older persons, but physical activity does not encompass all situations of social participation (52). Therefore, an independent examination of physical activity and social participation and their co-occurrence in the same study may provide a more comprehensive picture of health outcomes in older adults. This study examines for the first time the role of different levels of physical activity between digital technology use, social participation, and depression in older adults. Previous studies have found that certain factors influence the association between physical activity and depression. For example, it has been found that PA was associated with a lower risk of depression in males but not in females (21). Therefore, to control for the effects of other factors on physical activity, we similarly controlled for potential variables such as gender when analyzing this moderated mediation model. After moderating PAL, we found that there was a positive moderating effect on the association between digital technology use and social participation, which was negatively associated with depression. It was not influenced by gender. This may be because of the mediating role of social participation. Our results showed that all PAL positively moderated social participation. Notably, older women can better maintain their mental health and social participation during group physical activity (53). This finding supports our conclusions.

Surprisingly, in VPAL, digital technology use had the greatest positive impact on social participation and showed a significant gap from LPAL and MPAL, while the mediating role of social participation between digital technology use and depression was also enhanced with increasing PAL. Specifically, digital technology use was more likely to improve social participation and depression among older adults with VPAL. In this regard, we believe that older adults are more likely to have contact with new things and different people because of their relatively frequent physical activity, resulting in a smaller digital divide and greater social participation, leading to lower levels of depression. Physiologically, PA, such as exercise, can improve resistance to oxidative and physiological stress, stimulate several neuroplastic processes involved in depression, and promote self-esteem, social support and self-efficacy, which are important routes for physical activity to reduce depressive symptoms (54).

Previous studies have suggested that collective sports activities are not only an effective way for older adults to establish health and prevent disease progression but also a platform for older adults to rebuild social connections (55). In other words, older adults who participate in sports can meet more like-minded partners through their favorite sports and create frequent connections with people and society through the same hobbies and goals. Of course, older adults with high levels of physical activity are more likely to participate in various and diverse sports activities than those with medium or low levels of physical activity, rather than fulfilling daily essential physical activities such as household chores and commuting, thereby increasing their chances of connecting with the outside world through different media. Notably, in the Internet era, the use of digital devices has become an indispensable means of mass communication. They can use the Internet to establish more contacts with partners, expand social networks, make their contacts with society closer, and participate more in social participation (33). The increase in social participation can precisely contribute to reducing depression levels in older adults (56). This provides a basis for early prevention and psychological intervention of depression symptoms in older adults.

### Limitations

This study has some limitations. Firstly, it is a cross-sectional study and cannot explain the causal association between digital technology use and depression. In addition, physical activity was measured using self-reported questionnaires, and older adults often overestimated their PAL. Third, conducting cognitive assessments during the 2020 pandemic was challenging. Telephone interviews were used to measure cognitive scores in the study, which may have influenced participants’ ability to hear the interviewer or the interviewer’s ability to hear the participant.

## Conclusions

This study revealed the association between digital technology use and depression in older people, the partial mediating role of social participation between the two, and the moderating role of PAL in the association between digital technology use and social participation. In addition, the research findings highlight the positive effect of physical activity, especially VPAL, suggesting that this may be an important factor in improving social participation and preventing depression among older people. In light of these findings, older adults should be encouraged to integrate into digital life and actively pay attention to their physical activity, call on them to improve their PAL and increase their social participation. Governments can enhance the development of Internet infrastructure, disseminate knowledge about digital engagement among the elderly at the community level, and increase their Internet access. Concurrently, ensure the construction of basic public sports facilities and venues, such as activity centers for the elderly, to provide social and sports places for the elderly. These actions are crucial for preventing depression and maintaining mental health in older adults.

## Data Availability

The data that support the findings of this study are available from the China Health and Retirement Longitudinal Study (CHARLS)data repository (57).
