# Supplementary material for: The association between digital technology use and depression among older people in China: a moderated mediation model
Source: Front Psychiatry. 2025 Feb 10;16:1457967. doi: 10.3389/fpsyt.2025.1457967 (PMC11847860; doi:10.3389/fpsyt.2025.1457967)
Supplement: Supplementary file 1 [file DataSheet1.pdf]

Appendix.

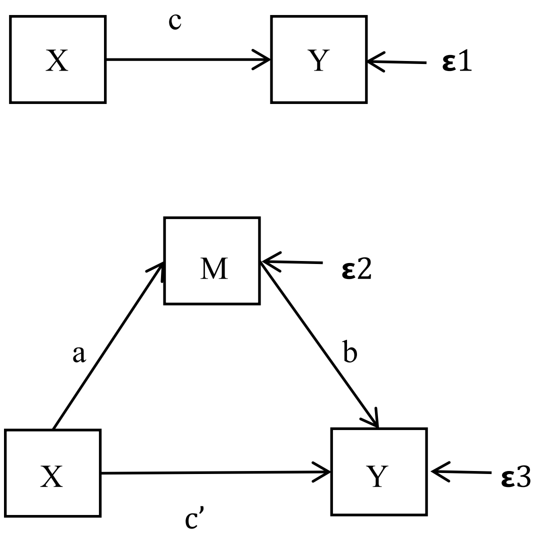

$$Y = cX + \varepsilon 1 \quad (1)$$

$$M = aX + \varepsilon 2 \quad (2)$$

$$Y = c'X + bM + \varepsilon 3 \quad (3)$$

Supplementary Figure a. Principle explanation of the mediation model construction.
(1) to obtain the total effect value c.
(2) to obtain direct effect value c’ and value b.
(3) to obtain the value a.
Mediator effect: a*b

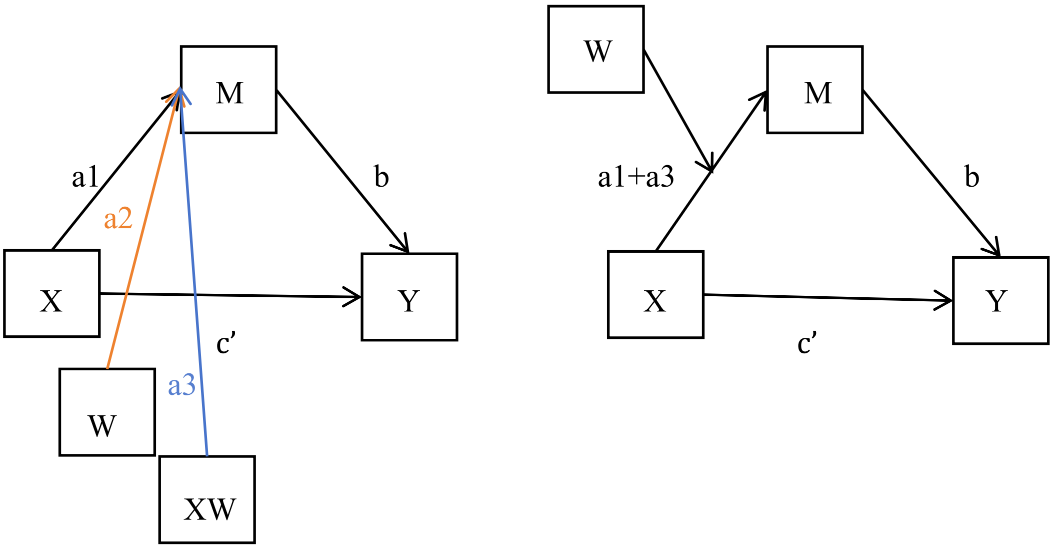

Supplementary Figure b. Principle explanation of the mediation model construction.
Moderated mediator effect: (a1+a3)*b

Supplementary Table. The subgroup analysis.

	DE		DD		DP	
Subgroups	OR (95%CI)	P	OR (95%CI)	P	OR (95%CI)	P
Gender						
Male (3,178)	0.664 (0.517, 0.853)	0.001	0.694 (0.554, 0.868)	0.001	0.913 (0.863, 0.965)	0.001
Female (2,566)	0.786 (0.622, 1.003)	0.053	0.791 (0.639, 0.977)	0.030	0.922 (0.873, 0.974)	0.004
Age (years)						
60-69 (3,899)	0.737 (0.608, 0.894)	0.002	0.750 (0.631, 0.891)	0.001	0.921 (0.881, 0.962)	$<$0.001
70-79 (1,598)	0.755 (0.506, 1.126)	0.168	0.753 (0.534, 1.060)	0.104	0.926 (0.844, 1.016)	0.104
≥80 (247)	1.404 (0.374, 5.277)	0.615	1.560 (0.503, 4.833)	0.441	1.028 (0.745, 1.418)	0.866
Chronic disease status						
No (3,654)	0.740 (0.591, 0.923)	0.008	0.017 (0.643, 0.958)	0.017	0.914 (0.868, 0.963)	0.001
1 type (1,418)	0.642 (0.456, 0.902)	0.011	0.614 (0.452, 0.835)	0.002	0.898 (0.831, 0.969)	0.006
≥2 types (672)	0.823 (0.523, 1.293)	0.398	0.799 (0.542, 1.177)	0.257	0.958 (0.869, 1.055)	0.380

DE, digital engagement. DD, digital devices. DP, digital purpose.
